# Pure laparoscopic radical nephroureterectomy for complicated renal pelvis carcinoma combined with horseshoe kidney: A case report and literature review

**DOI:** 10.3389/fonc.2022.1030626

**Published:** 2022-10-25

**Authors:** Ruizhen Huang, Jie Tian, Weifan Jiang

**Affiliations:** Department of Urology, The Second Affiliated Hospital of Nanchang University, Nanchang, China

**Keywords:** laparoscopic radical nephroureterectomy, renal pelvis carcinoma, horseshoe kidney, case report, surgery

## Abstract

We reported a case of pure laparoscopic radical nephroureterectomy for complicated renal pelvis carcinoma combined with horseshoe kidney (HSK). The aim was to present a case report and review of the literature about renal pelvis carcinoma combined with HSK. The case report includes a history of patient data. The pure laparoscopic radical nephroureterectomy was provided with the informed consent of the patient. A 53-year-old patient was diagnosed with a right renal pelvis mass with HSK. We performed laparoscopic radical nephroureterectomy with partial cystectomy and horseshoe renal isthmus amputation. Histopathological features, computed tomography urography (CTU), and angiography (CTA) confirmed the diagnosis of renal pelvis carcinoma combined with HSK. The tumor was removed, and the patient had an uneventful recovery. Renal pelvis carcinoma combined with HSK is a rare case. Due to severe anatomical abnormalities, this disease is a major challenge for urologists. We share our successful case for readers to learn from.

## Introduction

Horseshoe kidney (HSK) is a common type of congenital abnormality in renal fusion malformation, which is often regarded to occur in the fourth week of embryonic development. Epidemiological studies showed that it has a prevalence of approximately 0.25% in the general population, and male individuals seemed to be susceptible to suffering from this kind of deformities, with a male to female ratio of approximately 2:1 (1, 2). However, rare cases of renal carcinoma combined with HSK were reported, let alone the complex renal pelvis carcinoma complicated by HSK, which leads to big challenges for urologists in cancer management due to the complicated anatomical features. Herein, we present a case of complex renal pelvis carcinoma of the horseshoe kidney.

## Case presentation

A 53-year-old man presented with asymptomatic gross hematuria for half a year. He complained that there was no obvious cause for the hematuria half a year ago, and it was accompanied by frequent urination, urgency, and pain in urination. No attention was paid to this symptom. One day ago, the patient underwent abdominal B-ultrasound on physical examination, and a right renal pelvis tumor was identified; he thus visited our hospital for treatment.

**Figure 1:**
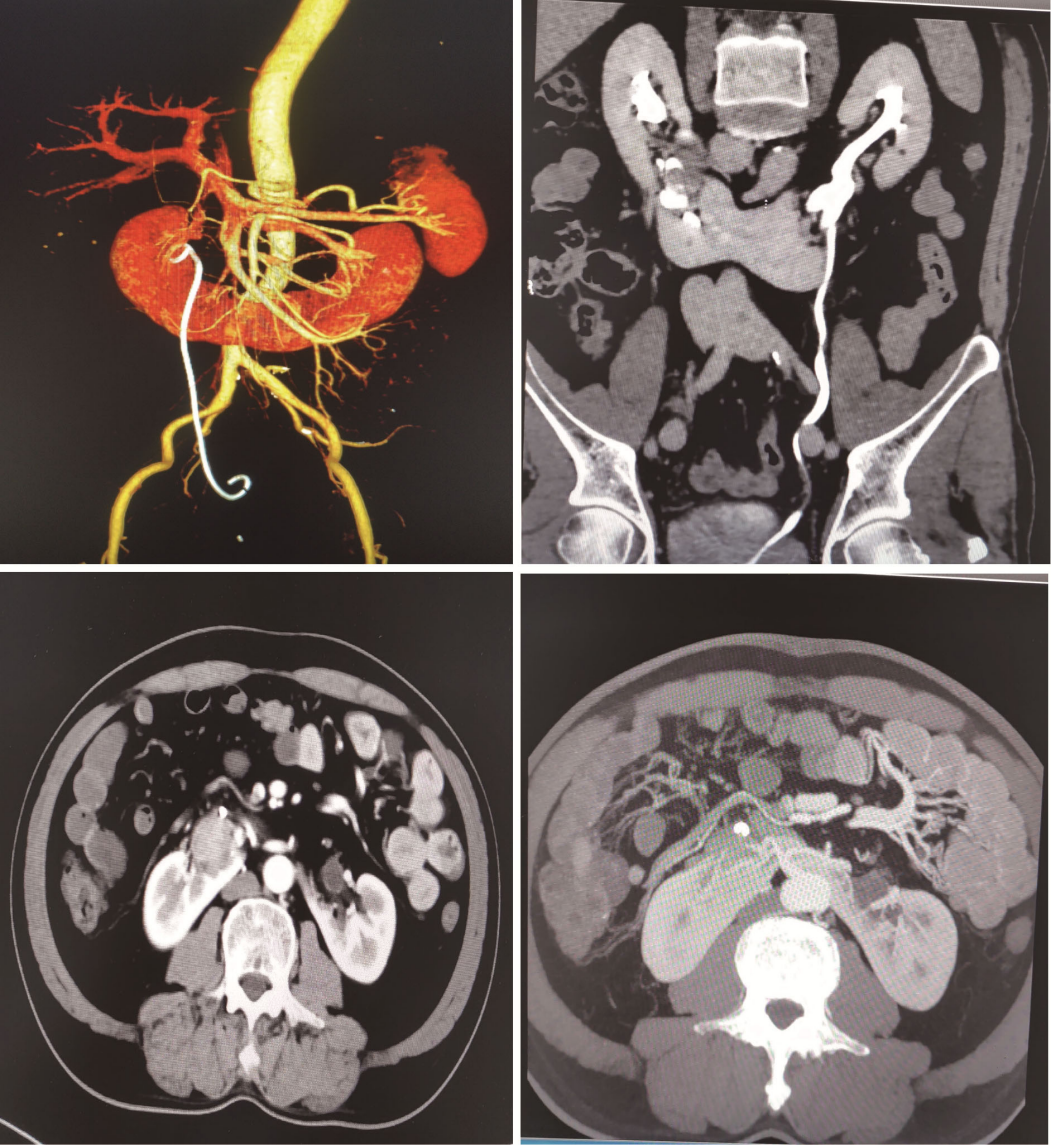
CTU and CTA show well-defined right renal pelvis mass complicated with horseshoe kidney and hydronephrosis.

Laboratory investigations showed that baseline hematological and biochemical investigations and urinalysis were normal. Glomerular filtration rate (GFR) results showed decreased blood perfusion in both kidneys and significantly impaired renal function (left kidney, 22.15 ml/min; right kidney, 18.25 ml/min; total, 40.40 ml/min). Preoperative imagological examination (**Figure 1**) demonstrated a right renal pelvis mass with hydronephrosis, horseshoe kidney, and ureteral stent placement in the right urinary tract.

**Figure 2:**
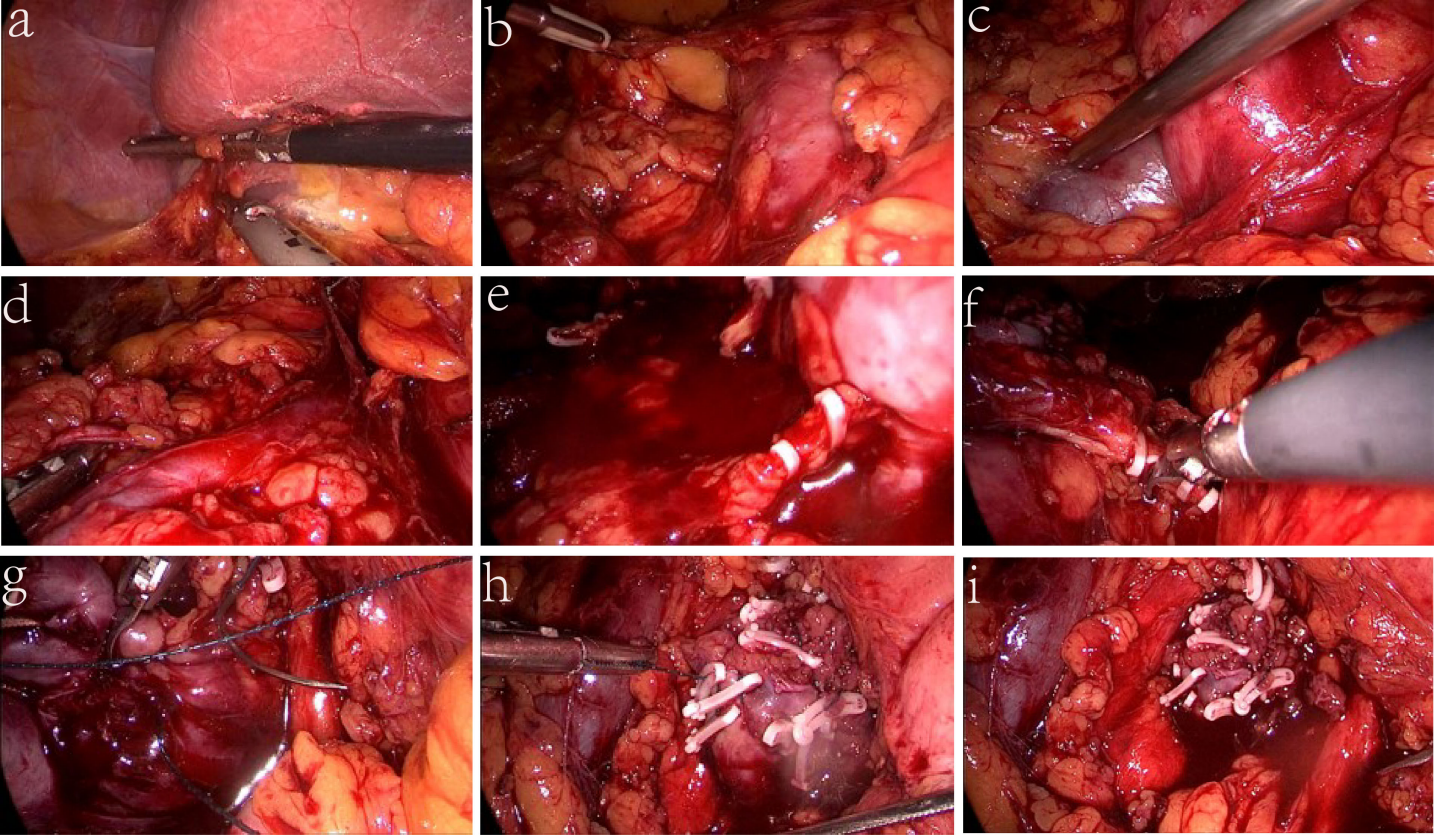
Key steps of the surgery. **(A)** Retracted the liver cephalad and exposed kidney. **(B)** Dissociated the ascending colon and duodenum to the midline. **(C)** Exposed the inferior vena cava. **(D)** Cut off the first renal artery and vein. **(E)** Divided the vasa vasorum. **(F)** Cut off the second renal artery and vein. **(G)** Fully exposed the isthmus and started slicing. **(H)** Sutured the left renal isthmus stump. **(I)** Sutured the bladder and lateral peritoneum.

The patient was diagnosed with a right renal pelvis mass complicated by HSK and underwent right transurethral ureteroscopic ureteral biopsy with ureteral stent placement on 14 June 2022. Postoperative pathology reported a high-grade non-invasive papillary urothelial carcinoma. Elective laparoscopic radical nephroureterectomy with partial cystectomy and horseshoe renal isthmus isthmusectomy were performed in the left decubitus position on 21 June 2022. We retracted the liver cephalad and incised the lateral peritoneum to expose the kidney (**Figure 2A**), then dissociated the ascending colon and duodenum to the ventral midline (**Figure 2B**). In the next step, we located the ureter and exposed the inferior vena cava behind the right kidney (**Figure 2C**). The fat and the fascia were carefully cleaned until the pulsating renal artery was visualized at the right renal hilum, then inspected and clipped with Hem-0-Lok clips (**Figure 2D**). The vasa vasorum were clamped, cauterized, and divided (**Figure 2E**), and the second renal artery and vein in the medial inferior part of the right kidney were cut off (**Figure 2F**). Finally, we fully exposed the isthmus and started slicing, using a 2-0 barbed thread to suture while slicing until the isthmus was completely dissected (**Figure 2G**), and then sutured the left renal isthmus stump properly (**Figures 2H, I** **)**. We lifted the right kidney, separated the intact right ureter and part of the entrance of the bladder, sutured the bladder with a 2-0 barbed thread, placed a closed system drain in the Gerota’s fascia and away from the renal parenchymal closure, and then closed the lateral peritoneum. The excised specimen was extracted by using a deployable entrapment sack from the abdominal space. The operating time was 4.5 h, and the estimated total blood loss was 200 ml.

**Figure 3:**
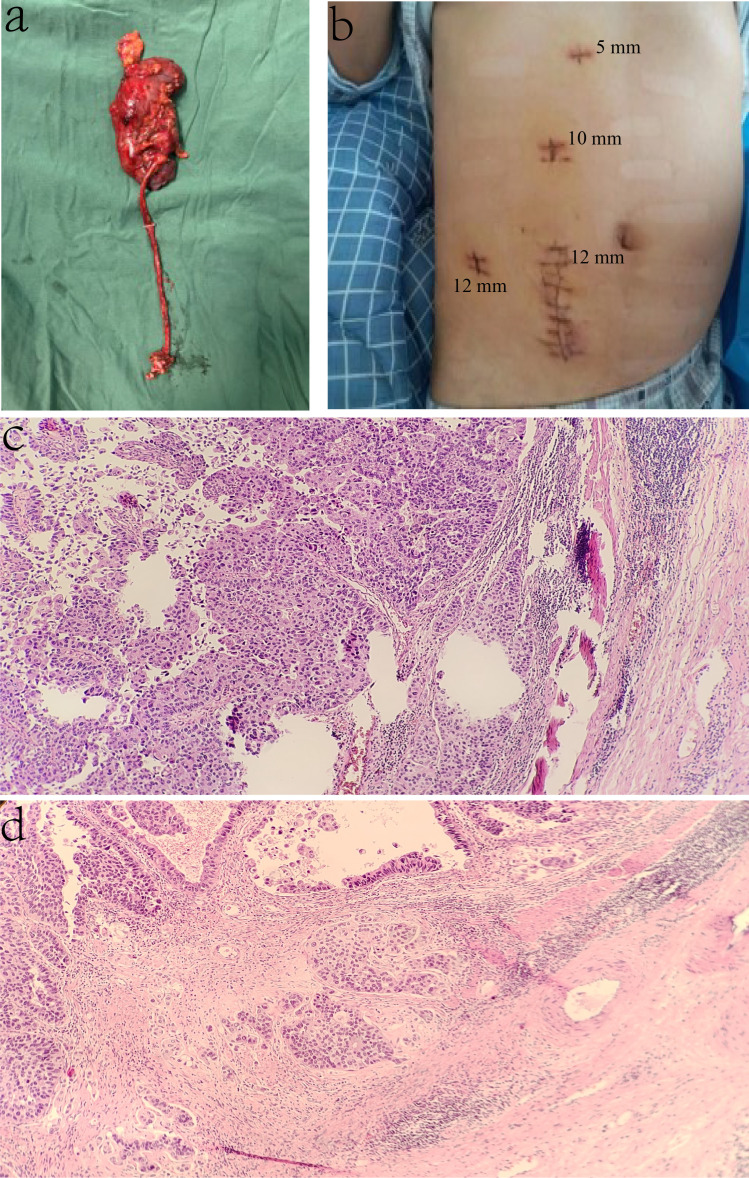
Cancer characteristics. **(A)** Gross appearance of the specimen; the tumor is 4 × 3 × 1.5 cm mass in the transition between the renal pelvis and ureter. **(B)** The Trocar port is placed during the tumor separation procedure. **(C, D)** The histopathological findings of the isolated specimen.

The gross appearance of the excised specimen showed a 13 × 4.5 × 3 cm kidney, a 20-cm-long ureter with a diameter of 0.7 cm, and part of the bladder wall of 2 × 1.5 × 1.3 cm. In addition, a 4 × 3 × 1.5 cm mass was seen at the transition of the renal pelvis and ureter (**Figure 3A**). The incision of the trocar healed well, and no intraoperative complications such as infection and bleeding occurred (**Figure 3B**). The histopathological findings of the renal pelvic tumor revealed high-grade invasive urothelial carcinoma, invading the muscularis mucosa of the renal pelvis. A tumor thrombus was seen in the vessel, and no nerve invasion was seen. There was no cancer involvement in the ureter and bladder wall (**Figures 3C, D** **)**. Epirubicin was given intravesical instillation after the operation, and the patient was discharged from the hospital 1 week after the operation. A recent follow-up occurred on 31 August 2022; the recovery was satisfying, and no apparent surgery-associated complications or recurrence were reported so far.

## Discussion

The formation of HSK may be the result of the fusion between the two umbilical arteries in the early embryonic stage (1). The anatomical abnormalities of the horseshoe kidney include several aspects: (1) anatomical variation of arteriovenous vessels, such as variable blood supply, and the variant blood vessels can be derived from the inferior mesenteric artery, abdominal aorta, contralateral renal artery, common iliac artery, and internal iliac artery; (2) both sides of the horseshoe kidney are connected by isthmus, which causes the ureter to be compressed when it passes through the ventral side of the isthmus; and (3) the HSK renal hilus turns ventrally by malrotation (3, 4). The abnormal anatomical position of the horseshoe kidney often causes poor urine drainage, which is an important reason for chronic urinary tract obstruction. Therefore, patients are prone to urinary calculi and infection in clinical practice. A comprehensive preoperative examination is necessary for treatment due to the existence of anatomical variations in the horseshoe kidney. The advantages of CTA and CTU in the reconstruction of the vascular and collecting systems may help us to make an adequate preoperative evaluation. For patients diagnosed with renal pelvis tumors complicated with HSK, surgical therapy should be recommended for those without contraindications.

To our knowledge, the radical complexity of managing horseshoe kidneys laparoscopically is the performance of isthmusectomy. Palmer et al. reported a case of urothelial carcinoma (UC) with HSK in which the application of the hot blade of the ultrasonic scalpel in isthmusectomy has unique advantages (5). In addition, it was said that the Endo GIA was useful in isthmusectomy, which is fast and convenient. No supplementary sutures were needed, and no more than 1 min was spent in the isthmus section, which may, in other words, bring many benefits to patients (6). In our presented case, the isthmus was thicker than usual. Because it is intractable to perform this operation, we used the method of suturing while resecting the isthmus, and without the employment of bulldog clamps or forceps, HSK isthmus division was completed appropriately. In addition, to avoid the cancer cells implanted along the right ureter, we ligated the upper ureter with Hem-o-lok during the procedure, which is especially worth noting. Comprehensive preoperative preparations and correct surgical planes are necessary for this kind of patient; the three-dimensional analyzer of the images and intraoperative indocyanine green (ICG) fluorescence system may help us further confirm the blood supply (7), which may be useful in nephron preservation without compromising oncological outcomes. We suggest that the use of laparoscopic HSK surgery will cause minimal surgical insult to patients and effectively improve the patient’s quality of life. We will strive to improve our professional abilities for better medical service.

## Data availability statement

The original contributions presented in the study are included in the article/supplementary material. Further inquiries can be directed to the corresponding author.

## Ethics statement

This study involving human participants was reviewed and approved by the Ethics Committee of the Second Affiliated Hospital of Nanchang University. The patients/participants have provided their written informed consent to participate in this study

## Author contributions

RH and JT analyzed and interpreted the patient data and drafted the paper. RH and JT contributed equally to this work. WJ conceptualized the study and design, analysis, and interpretation of data. All authors contributed to the article and approved the submitted version.

## Funding

Grants from the Grants from the China National Natural Science Foundation (No. 82160579) and Jiangxi Provincial Natural Science Foundation Project (No. 20192BAB205026) supported this study. The funders had no role in this study.

## Conflict of interest

The authors declare that the research was conducted in the absence of any commercial or financial relationships that could be construed as a potential conflict of interest.

## Publisher’s note

All claims expressed in this article are solely those of the authors and do not necessarily represent those of their affiliated organizations, or those of the publisher, the editors and the reviewers. Any product that may be evaluated in this article, or claim that may be made by its manufacturer, is not guaranteed or endorsed by the publisher.
